# Andrographolide binds to spike glycoprotein and RNA-dependent RNA polymerase (NSP12) of SARS-CoV-2 by in silico approach: a probable molecule in the development of anti-coronaviral drug

**DOI:** 10.1186/s43141-021-00201-7

**Published:** 2021-07-13

**Authors:** Lokanathan Srikanth, Potukuchi Venkata Gurunadha Krishna Sarma

**Affiliations:** Department of Biotechnology, Sri Venkateswara Institute of Medical Sciences and University, Tirupati, Andhra Pradesh 517507 India

**Keywords:** Andrographolide, RBD, NSP12, ACE 2 receptor, SARS-CoV-2

## Abstract

The SARS-CoV-2 belongs to Coronaviridae family infects host cells by the interaction of its spike glycoprotein and angiotensin-converting enzyme 2 (ACE 2) of host cells. Upon entry, the virus uses its RNA dependent RNA polymerase (NSP12) for transcribing its genome to survive in the cell and spread its infection. The protein sequences of receptor-binding domain (RBD) of spike glycoprotein, and NSP12 exhibits high homology in the family of Coronoviridae and are ideal candidates for the development of anti-coronaviral drugs. In the quest to identify inhibitory molecules against these proteins, we searched several molecules that are present in naturally occurring medicinal plants database. Andrographolide which is largely present in the leaf extracts of *Andrographis paniculata* (AP) and is known to exhibit antiviral, antibacterial, and stabilizes Th1/Th2/Th17 responses; taking this clue, we used in silico approaches to see the binding of andrographolide to RBD and NSP12 molecules. Our docking results showed very strong affinity of andrographolide to RBD and NSP12 of the SARS-CoV-2 virus with dock scores of −10.3460 for RBD and −10.7313 for NSP12 indicating andrographolide acts as an inhibitor of RBD and NSP12. These unique properties of andrographolide, AP extract, can be tested as anti-coronaviral drug.

## Introduction

The pandemic caused by SARS-CoV-2 which has emerged from China has devastated whole humanity. The genome of SARS-CoV-2 and several viruses belonging to Coronaviridae family (severe acute respiratory syndrome-associated coronavirus (SARS-CoV) and Middle East respiratory syndrome-associated coronavirus (MERS-CoV)) have been sequenced and annotated. The genome sequence similarity of SARS-CoV-2 confirms it is a beta coronavirus [1, 2]. It appears that spike glycoprotein expressed on the surface of SARS-CoV-2 is responsible facilitating the entry of the virus into the host cells, this is the resultant of strong binding with angiotensin-converting enzyme 2 (ACE 2) of the host cells [3, 4]. This SARS-CoV-2 infection disturbs the host immune system with elevated Th17 response [5]; this is where people with low immunity are prone for fatal consequences.

Characterization of spike glycoprotein is important in understanding its mode of entry and in the development of therapeutics against SARS-CoV-2. All coronaviruses enter into host cells utilizing spike glycoprotein which gives coronaviruses a crown-like appearance by forming spikes on their surface. The analysis of spike glycoprotein amino acid sequence shows a large ectodomain, a single-pass transmembrane anchor, and a short C-terminal intracellular tail [6]. The ectodomain includes a receptor-binding unit S1 and a membrane-fusion unit S2. The results of electron microscope showed spike glycoprotein consists of a clove-shaped spike with three S1 heads and a trimeric S2 stalk. The entry of virus to host cell is facilitated by S1 which binds to the ACE 2 through receptor-binding domain (RBD), while S2 fuses host cell membrane with viral envelope allowing the viral genome to enter into host cell. Specific RBD-receptor binding determines if a cell can be infected and also serves as a target for therapeutic developments to treat diseases caused by coronaviruses [7, 8].

This SARS-CoV-2 infection to the host cells, coronaviruses utilizes a multi-subunit RNA-synthesis complex of viral non-structural proteins (NSP) responsible for the replication and transcription of the viral genome. The SARS-CoVNSP12 polymerase is associated with two other essential proteins, NSP7 and NSP8. The N-terminal of NSP12 contains a common structure which is conserved in all coronaviral polymerases as a large structure having kinase-like fold bound by two NSP8 molecules. This demonstrates NSP12 complex a potent therapeutic target in the development anti-coronaviral drugs [9].

The 7.27 million confirmed cases of SARS-CoV-2 and mortality over 413,000 as of 12 June, 2020, has made all the countries to search for effective therapeutic modalities in the treatment of COVID-19. One of the approved lists of drugs in the treatment of COVID-19 is remdesivir; it inhibits NSP 12 and thereby prevents RNA synthesis and kills the virus. This remdesivir molecular formula is C_27_H_35_N_6_O_8_P; the structure appears to have prominent naphthalene ring [9]. The remdesivir treatment has side effects on the liver and kidney functions and this is an FDA-approved drug in the treatment of COVID-19; earlier, it was used in the treatment of Ebola infection [10, 11].

In India and other parts of Asia, large number of medicinal plant extracts and their derivatives are largely used in the treatment of various ailments including viral diseases. Andrographolide a major constituent present in the leaf extract of *Andrographis paniculata* (AP) is used as herbal medicine which possesses antiviral, antibacterial, anti-inflammatory effects, and stabilizes Th1/Th2/Th17 responses [12–14]. The chemical name of andrographolide is 3α, 14, 15, 18-tetrahydroxy-5β, 9βH, 10α-labda-8, 12-dien-16-oic acid γ-lactone, and its molecular formula is C_20_H_30_O_5_. The structure of andrographolide has been analyzed by using X-ray, 1H,13 C-NMR, and ESI-MS [15]. Even though andrographolide is not very soluble in water, it is soluble in acetone, chloroform, ether, and hot ethanol. Crystalline andrographolide reported to be highly stable up to 3 months [15]. The structure of remdesivir and andrographolide has one common naphthalene ring, and andrographolide is smaller than remdesivir [9, 15]. These evidences encouraged us to comprehend the anti-SARS-CoV-2 effect of andrographolide. In this study, we used in silico approach to dock andrographolide structure to RBD and NSP12 protein structures of SARS-CoV-2 to predict that AP extract can be used for anti-coronaviral treatment.

## Methods

Protein sequences of RBD and NSP12 of SARS-CoV-2 were compared and multiple sequence alignment (MSA) was performed by using the Clustal-W program. The phylogenetic analysis of spike RBD’s of SARS-Cov-2 (PDB ID: 6LZG) compared with SARS (2AJF), MERS (4L72), HKU4 (4QZV), and HKU1 (5KWB). Among the NSP12, i.e., RNA-dependent RNA polymerase(RdRP) of SARS-CoV-2 reported at various countries like Australia (QJR96151.1), Belgium (QIB84671.1), France (QJT73032.1), Guangzhou (China) (QJQ84086.1), India (QJQ28427.1), Japan (BCF74567.1), and the USA (QJP03561.1) were analyzed with MSA followed by super imposing their structures being built by using Modeller 9.24 taking the three-dimensional structure of PDB ID: 6M71 as template. Molecular docking of andrographolide to SARS-CoV-2 spike RBD and NSP12 (RdRP) structures were performed using Autodock and MOE softwares.

## Results

### Analysis of spike glycoprotein of SARS Cov-2

**Fig. 1:**
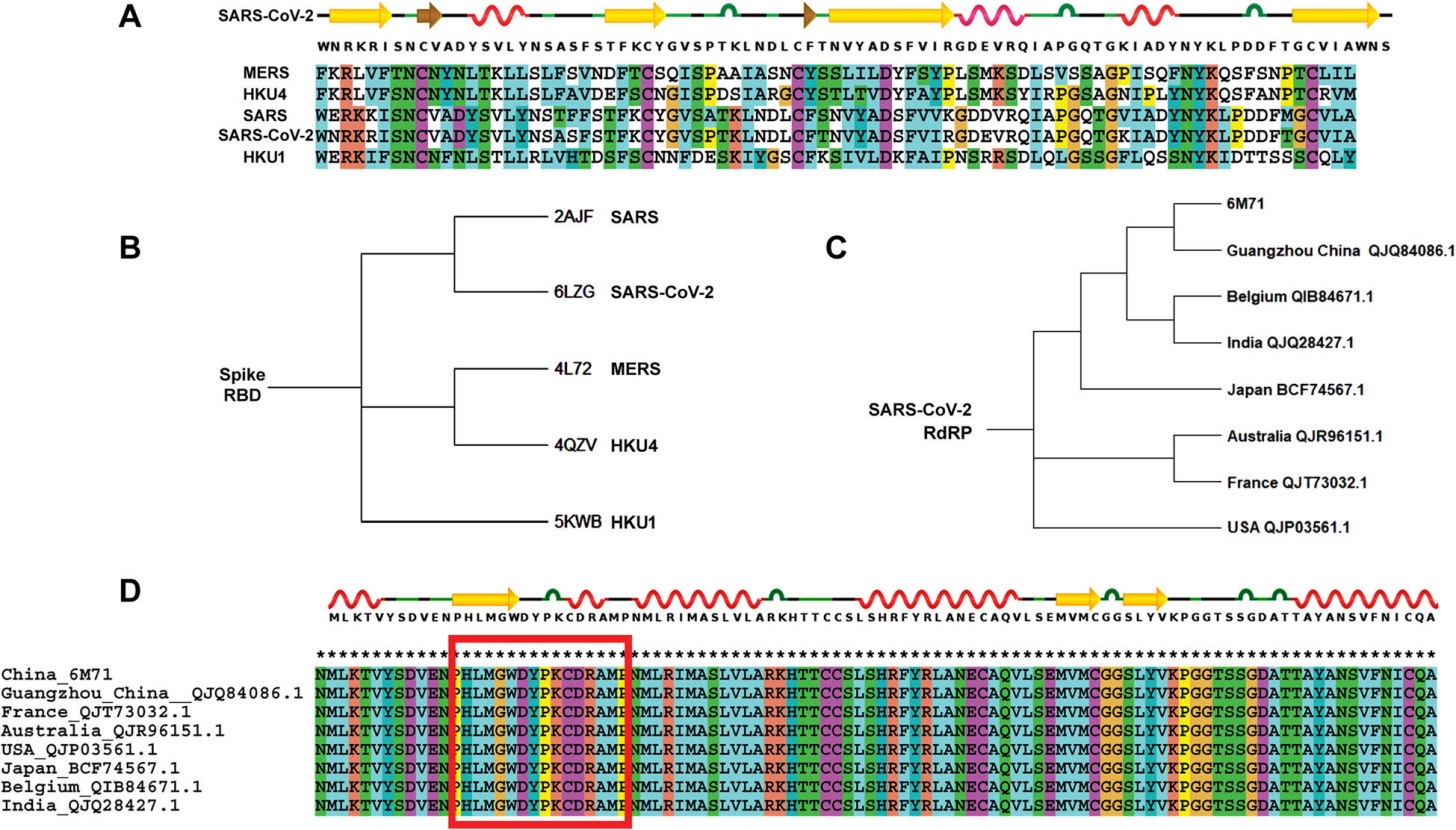
Comparison of SARS-CoV-2 with closely related coronaviruses. **A** Amino acid sequence alignment for RBD of spike glycoprotein from SARS-CoV-2 and its closely related coronaviruses. **B** Phylogenetic analysis of related coronaviruses based on spike receptor-binding domain (RBD). **C** Phylogenetic analysis of RNA-dependent RNA polymerase (RdRP) of SARS-CoV-2 from different countries. **D** Amino acid sequence alignment for RdRP from SARS-CoV-2 and its contemporary strains present globally

**Fig. 2:**
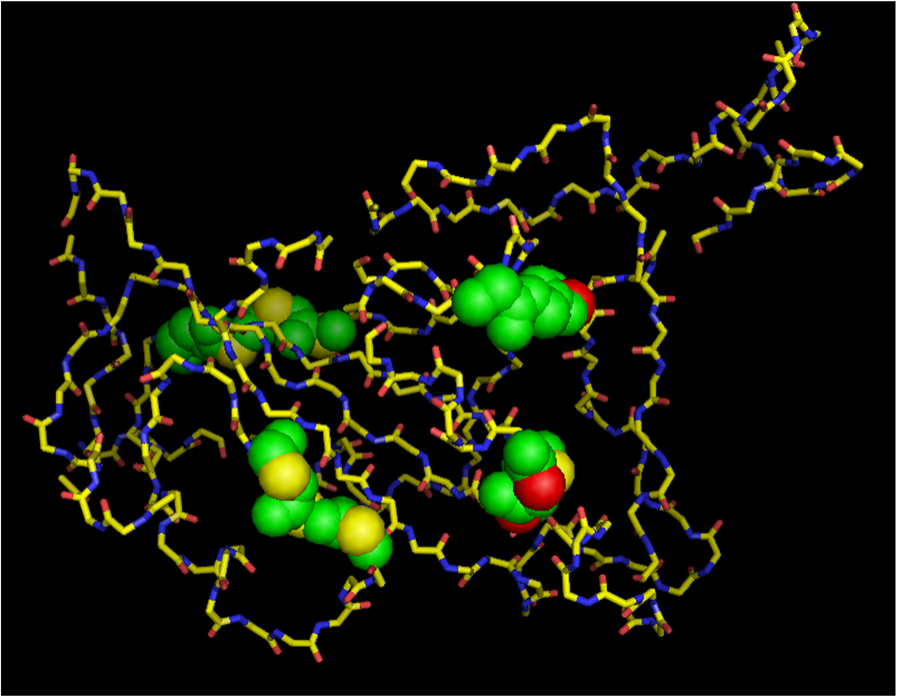
Kinases/phosphorylation sites of spike receptor-binding domain (RBD). The motifs (spheres) on spike RBD domain (sticks) determined from Scan Prosite revealed cAMP- and cGMP-dependent protein kinase phosphorylation site: “KRIS;” protein kinase C phosphorylation site: “TFK” and “TGK;” cell attachment sequence: “RGD”

The findings of MSA for spike glycoprotein with other members of coronavirus (SARS, MERS, HKU4, and HKU1) indicate conserved S1 and S2 domains which are the feature in spike protein of Coronaviridae family; however, the RBD region of coronavirus is highly variable which makes it defensive to bind and enter the host cells (Fig. 1A, B). This unpredictable protein sequence makes difficult to produce vaccines against it. This spike protein also possesses unique Zinc-binding region. The conspicuous presence of Y and S/T phosphorylating sites as predicted from NetPhos 3.1 server explains the functional role of spike protein in SARS-CoV-2. Presence of these sites on the spike protein (Fig. 2) probably stimulates tyrosine kinases and S/T kinases making the host cell metabolically active and thereby increasing the rate of proliferation.

### RNA dependent RNA polymerase NSP12, NSP7, and NSP8 structure analysis

The sequence analysis of NSP12, NSP7, and NSP8 of SARS-CoV-2 (PDB ID: 6M71) reveal all three NSP12, NSP7, and NSP8 are present together in this multicomplex enzyme. The sequence analysis reveals presence of polymerase activity confined to NSP12 while kinase activity was observed in NSP8 and this is the common feature in all coronaviruses. Majority of the strains reported globally were aligned phylogenetically for NSP12 protein sequence (Fig. 1C), and the MSA revealed there were large areas of conserved regions in which our docking targeted site becomes universal for SARS-CoV-2 that reported by various countries with 100% identity (Fig. 1D).

### Homology modeling for RdRP structures

**Fig. 3:**
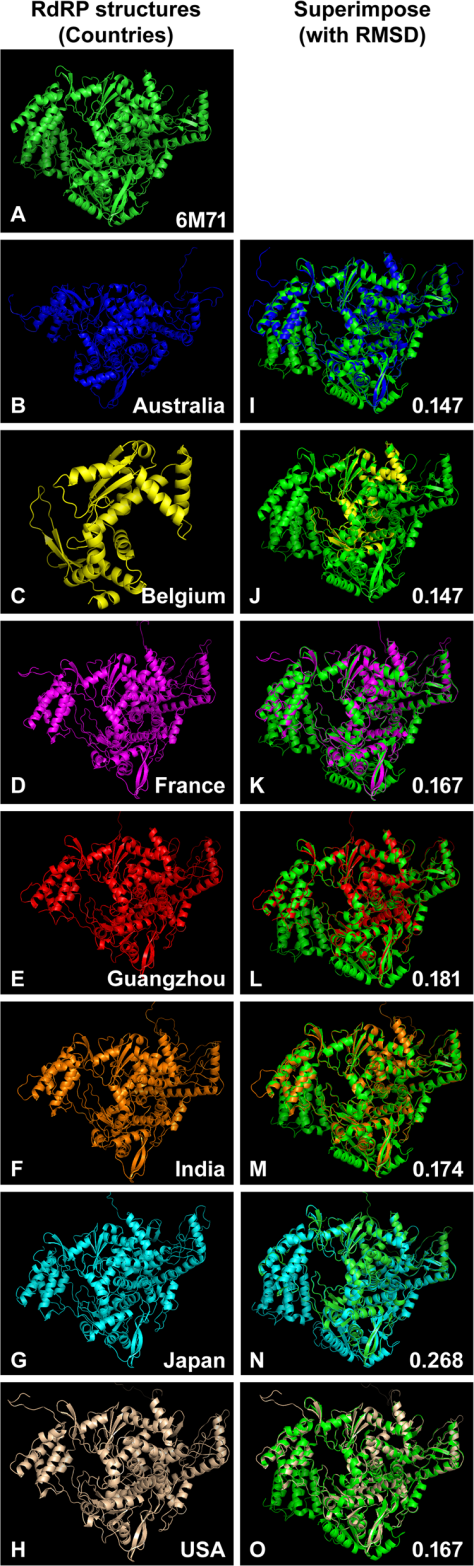
Superimposition and comparison of RdRP structures of SARS-CoV-2. **A** Three-dimensional structure of 6M71 as template. **B** RdRP structures built with Modeller 9.24 for Australia (QJR96151.1). **C** Belgium (QIB84671.1). **D** France (QJT73032.1). **E** Guangzhou (China) (QJQ84086.1). **F** India (QJQ28427.1). **G** Japan (BCF74567.1). **H** USA (QJP03561.1). **I** Superimposition of 6M71 on built structures of Australia, **J** Belgium, **K** France, **L** Guangzhou, **M** India, **N** Japan, **O** USA with their corresponding RMSD values

With this highly conserved and consistent sequence of RdRP were used to target to cease the replicational activities inside the host. For this, the docking efficiency needs to be tested after these three-dimensional structures were built using the Modeller 9.24 software. The built structures were superimposed with template 6M71 and their RMS score were determined (Fig. 3).

### Docking of andrographolide to spike glycoprotein of SARS-CoV-2

Andrographolide is the major compound present in the leaf extract of *Andrographis paniculata* (AP). The molecular formula of andrographolideis C_20_H_30_O_5_ (PubChem CID: 5318517) is a lactone; this binds to the spike glycoprotein in the tyrosine kinase phosphorylating sites indicating inactivation of spike glycoprotein. Additionally, “Lipinski rule of 5” extricate drug-like molecules from non-drug like molecules in which this andrographolide has high probability of success due to drug likeness complying with the rules (Table 1).

**Table 1:** Lipinski rule of 5: Determination of drug likeliness of andrographolide

S No	Parameters	Prerequisite	Andrographolide	Result
**1**	**Molecular mass**	Less than 500 Dalton	350 Dalton	Pass
**2**	**High lipophilicity (expressed as LogP)**	Less than 5	1.9626	Pass
**3**	**Hydrogen bond donors**	Less than 5	3	Pass
**4**	**Hydrogen bond acceptors**	Less than 10	5	Pass
**5**	**Molar refractivity**	Between 40 and 130	93.560364	Pass

**Fig. 4:**
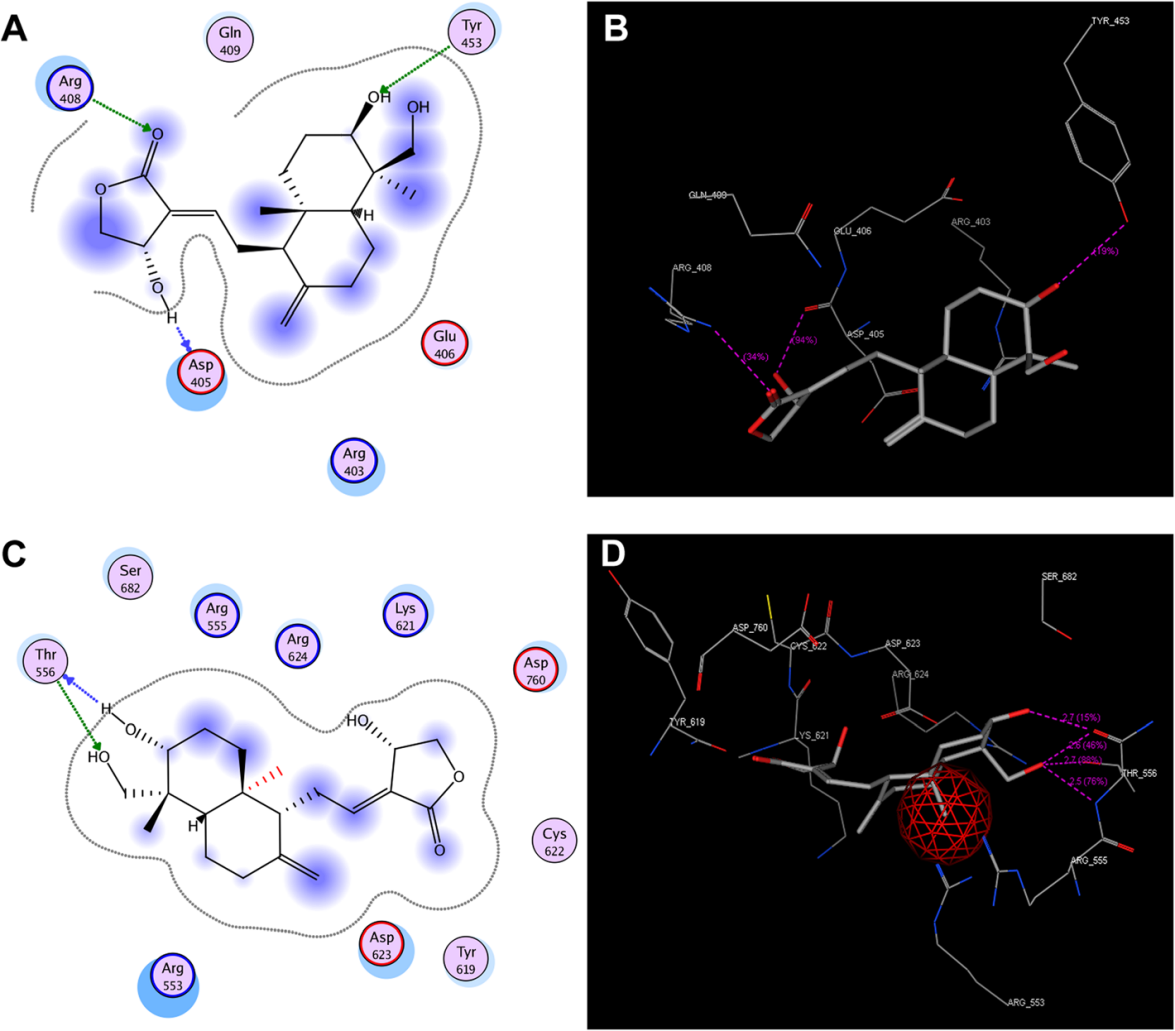
Docking of andrographolide with spike RBD and RdRP (NSP12) of SARS-CoV-2. **A** Spike RBD acting as a receptor to dock andrographolide projected 2-dimensionally. **B** Spike RBD 3-dimensional field of image showing interacting residues and the H-bonds. **C** 2-Dimensional portrait of RdRP (NSP12) receptor docked with andrographolide. **D** 3-Dimensional representation of RdRP (NSP12) interacting residues and its H-bonds

As mentioned earlier, the active docking site for andrographolide was targeted to kinases rich region present in the RBD sequence of 6LZG chain-2. The andrographolide binds to spike glycoprotein through 3 hydrogen bonds that are interacting with ASP405, ARG408, and TYR453 residues (Fig. 4A, B). The docking score was found to be −10.3460.

### Docking of andrographolide to NSP12 of SARS-CoV-2

The andrographolide docks very effectively in the active site present in the RdRP (-PHLMGWDYPKCDRAMP-) of NSP12 thereby preventing the RNA-dependent RNA polymerase activity. This function is essential for the virus to survive in the host and spread its infection. Andrographolide binds with THR556 residue by forming 4 hydrogen bonds; the binding mode reveals it is mostly competitive binding only (Fig. 4C, D). The docking score of −10.7313 was also much appreciable (Table 2).

**Table 2:** Docking scores of andrographolide with SARS-CoV-2 spike RBD and with RNA-dependent RNA polymerase of SARS-CoV-2

	Dock score	H-bonds	Interacting residues	H-bond length	H-bond score
**Spike RBD**	−10.3460	3	ASP 405	2.4 Å	34%
			ARG 408	2.8 Å	94%
			TYR 453	3.0 Å	19%
**RdRP**	−10.7313	4	THR 556	2.7 Å	15%
			THR 556	2.6 Å	46%
			THR 556	2.7 Å	88%
			THR 556	2.5 Å	76%

All these findings suggest that andrographolide can be a potent molecule that can inhibit the entry of SARS-CoV-2 virus in to the host cell at the same time it can prevent the amplification of the viral genome by binding to NSP12. This andrographolide is the active molecule present in AP extract which can be tested for anti-coronaviral treatment.

## Discussions

The transmittable infection of SARS-CoV-2 first appeared in Wuhan, China, which can spread from human to human even before persons become symptomatic has devastated whole globe [1, 2]. The spike glycoprotein of this new coronavirus SARS-Cov-2 binds to ACE2 and this interaction is the responsible entry into the host cells [1, 6, 7]. The RBD of spike glycoprotein of SARS-Cov-2 shares no structural homology with that of reported NL63-CoV but recognizes the same region in ACE2. However, RBD of NL63-CoV exhibits very low binding with ACE2, involving less amino acids [16]. This most probably results in a weaker interaction; it is however well known that NL63-CoV only causes mild to moderate respiratory infections and very less aggressive in terms of its spread in the population. The spike glycoprotein of SARS-CoV-2 shows high homology with all the other coronaviruses [8], having Y phosphorylation sites which has the ability to activate host tyrosine kinases such as various JAKs. It is known that JAKs phosphorylate STAT molecules and initiates expression of various cytokines in the host cells and very high expression of cytokines is observed in the serum of SARS-CoV-2-infected patients disrupting the immunity of the patients [5]. In the present study, the successful docking of andrographolide with RBD of spike glycoprotein of SARS-CoV-2 (Fig. 4B) this function of andrographolide presumably prevents spike glycoprotein interaction with ACE 2, thereby preventing the entry of SARS Cov-2 into the host cells.

Our findings also showed andrographolide binds in active site of NSP12 of SARS-CoV-2, thereby curtailing the NSP12 function. This interaction can prevent propagation of SARS-CoV-2 in the host cells (Fig. 4D). It has been observed that remdesivir inhibits NSP12 function if SARS-CoV-2 and is used as drug in the treatment of COVID-19; however, remdesivir treatment causes acute kidney and liver injuries [9–11]. As compared to the remdesivir andrographolide is much smaller molecule occurs naturally in the AP extract [12–14].

SARS-CoV-2 infection is associated with a cytokine squall, manifesting elevated serum levels of IL-1β, IL-2, IL-7, IL-8, IL-9, IL-10, IL-17, G-CSF, GM-CSF, IFNγ, TNFα, IP10, MCP1, MIP1A, and MIP1B [5].This andrographolide is also known to inhibit Th1/Th17 response and stabilizes the excessive expression of cytokines in the host [12–14]. In summary, andrographolide is the major constituent of AP extract inhibits entry of SARS-CoV-2 into the host cells by binding to the spike glycoprotein and also prevents propagation of SARS-CoV-2 in the host cells by blocking NSP12. Further, andrographolide is known to stabilize the cytokine storm observed in COVID-19 patients; also having number of beneficial effects, this AP extract can be used in the treatment of COVID-19 [17]. The concept of developing vaccines at an unprecedented speed including clinical trials and its perceptions added from studying the antibody features that associate with retrieval as opposed to worsening of disease will inform to rethink the type of antibodies used to evaluate in vaccine studies [18]. Since, we must not have been ignored on safety evaluation of candidate vaccines which in case of andrographolide have an advantage being potent drug rather than un-safety vaccines till these desperate situations.

## Data Availability

Not applicable

## Abbreviations

NSP: Non-structural proteins
SARS-CoV-2: severe acute respiratory syndrome coronavirus 2
ACE 2: Angiotensin-converting enzyme 2
RBD: Receptor-binding domain
MERS-CoV: Middle East respiratory syndrome-associated coronavirus
C-NMR: Carbon-13 nuclear magnetic resonance
ESI-MS: Electrospray ionization mass spectroscopy

## References

[1] Zhou P, Yang XL, Wang XG, Hu B, Zhang L, Zhang W, Si HR, Zhu Y, Li B, Huang CL, Chen HD, Chen J, Luo Y, Guo H, Jiang RD, Liu MQ, Chen Y, Shen XR, Wang X, Zheng XS, Zhao K, Chen QJ, Deng F, Liu LL, Yan B, Zhan FX, Wang YY, Xiao GF, Shi ZL (2020). A pneumonia outbreak associated with a new coronavirus of probable bat origin. Nature.

[2] Wu F, Zhao S, Yu B, Chen YM, Wang W, Song ZG, Hu Y, Tao ZW, Tian JH, Pei YY, Yuan ML, Zhang YL, Dai FH, Liu Y, Wang QM, Zheng JJ, Xu L, Holmes EC, Zhang YZ (2020). A new coronavirus associated with human respiratory disease in China. Nature.

[3] Luan J, Jin X, Lu Y, Zhang L (2020) SARS-CoV-2 spike protein favors ACE2 from Bovidae and Cricetidae [published online ahead of print, 2020 Apr 1]. J Med Virol. 10.1002/jmv.25817 10.1002/jmv.25817 PMC7228376 32239522

[4] Ortega JT, Serrano ML, Pujol FH, Rangel HR (2020). Role of changes in SARS-CoV-2 spike protein in the interaction with the human ACE2 receptor: an in silico analysis. EXCLI J.

[5] Wu D, Yang XO (2020). TH17 responses in cytokine storm of COVID-19: an emerging target of JAK2 inhibitor Fedratinib. J Microbiol Immunol Infect.

[6] Li F (2016). Structure, function, and evolution of coronavirus spike proteins. Annu Rev Virol.

[7] Andersen KG, Rambaut A, Lipkin WI, Holmes EC, Garry RF (2020). The proximal origin of SARS-CoV-2. Nat Med.

[8] Chen Y, Guo Y, Pan Y, Zhao ZJ (2020). Structure analysis of the receptor binding of 2019-nCoV. Biochem Biophys Res Commun.

[9] Kirchdoerfer RN, Ward AB (2019). Structure of the SARS-CoV nsp12 polymerase bound to nsp7 and nsp8 co-factors. Nat Commun.

[10] Scavone C, Brusco S, Bertini M et al (2020); [published online ahead of print, 2020 Apr 24]) Current pharmacological treatments for COVID-19: what’s next? Br J Pharmacol. 10.1111/bph.15072 10.1111/bph.15072 PMC7264618 32329520

[11] Beigel JH, Tomashek KM, Dodd LE et al (2020) [published online ahead of print, 2020 May 22) Remdesivir for the treatment of Covid-19 - preliminary report. N Engl J Med. 10.1056/NEJMoa2007764 10.1056/NEJMc2022236 32649078

[12] Jayakumar T, Hsieh CY, Lee JJ, Sheu JR (2013). Experimental and clinical pharmacology of Andrographis paniculata and its major bioactive phytoconstituent andrographolide. Evid Based Complement Alternat Med.

[13] Nie X, Chen SR, Wang K, Peng Y, Wang YT, Wang D, Wang Y, Zhou GC (2017). Attenuation of innate immunity by andrographolide derivatives through NF-κB signalling pathway. Sci Rep.

[14] Zhu Q, Zheng P, Zhou J, Chen X, Feng Y, Wang W, Zhou F, He Q (2018). Andrographolide affects Th1/Th2/Th17 responses of peripheral blood mononuclear cells from ulcerative colitis patients. Mol Med Rep.

[15] Rajani M, Shrivastava N, Ravishankara MN (2000). A rapid method for isolation of andrographolide from Andrographis paniculata Nees (kalmegh). Pharm Biol.

[16] Wu K, Li W, Peng G, Li F (2009). Crystal structure of NL63 respiratory coronavirus receptor-binding domain complexed with its human receptor. Proc Natl Acad Sci U S A.

[17] Ang L, Lee HW, Choi JY, Zhang J, Soo Lee M (2020). Herbal medicine and pattern identification for treating COVID-19: a rapid review of guidelines. Integr Med Res.

[18] Iwasaki A, Yang Y (2020). The potential danger of suboptimal antibody responses in COVID-19. Nat Rev Immunol.

